# Geophysical imaging of ophiolite structure in the United Arab Emirates

**DOI:** 10.1038/s41467-020-16521-0

**Published:** 2020-05-29

**Authors:** M. Y. Ali, A. B. Watts, M. P. Searle, B. Keats, S. Pilia, T. Ambrose

**Affiliations:** 1 Khalifa University of Science and Technology, Abu Dhabi, UAE; 2 Department Earth Sciences, University of Oxford, South Parks Road, Oxford, OX1 3AN UK; 3 Department of Earth Sciences, University of Cambridge, Cambridge, CB3 OEZ UK

**Keywords:** Structural geology, Geophysics, Tectonics

## Abstract

The Oman-United Arab Emirates ophiolite has been used extensively to document the geological processes that form oceanic crust. The geometry of the ophiolite, its extension into the Gulf of Oman, and the nature of the crust that underlies it are, however, unknown. Here, we show the ophiolite forms a high velocity, high density, >15 km thick east-dipping body that during emplacement flexed down a previously rifted continental margin thereby contributing to subsidence of flanking sedimentary basins. The western limit of the ophiolite is defined onshore by the Semail thrust while the eastern limit extends several km offshore, where it is defined seismically by a ~40–45°, east-dipping, normal fault. The fault is interpreted as the southwestern margin of an incipient suture zone that separates the Arabian plate from in situ Gulf of Oman oceanic crust and mantle presently subducting northwards beneath the Eurasian plate along the Makran trench.

## Abstract (web summary)

The Semail ophiolite provides evidence for geological processes that form oceanic crust, however, its deep structure remains debated. Here, the authors use geophysical imaging to determine that the ophiolite is bound by a thrust fault in the west, and a normal fault in the east, bounding a rapidly subsiding basin, implying the ophiolite may not be rooted in the Gulf of Oman crust.

## Introduction

Ophiolites, which comprise oceanic crust and mantle that have been thrust onto previously rifted continental margins prior to continental collision, are a key component of the Wilson cycle, a fundamental feature of which is the closing of ocean basins and the formation of mountains belts. The world’s largest and best known ophiolite is found in the Oman–United Arab Emirates (UAE) mountains^1–3^, a 1–3 km high, 700 km long by 150 km wide mountain belt now part of the Arabian plate. Here, approximately 150 Myr of rifted margin sedimentation ended abruptly during the Cenomanian–Turonian (~99–89 Ma) with collapse of the margin and development of a flanking foreland basin^4–8^ that accommodated the SW emplacement of thin-skinned thrust sheets of proximal–distal Tethyan sedimentary rocks (Hawasina–Haybi complexes), and a giant thrust sheet, the Semail ophiolite. The ophiolite, which formed between 96.5 and 95.0 Ma at a spreading centre above an active NE-dipping subduction zone^9,10^, comprises oceanic crustal and mantle rocks that were transported laterally at least 200 km, probably over 450 km in total, and the entire obduction history spanned ~27 Myr (from 95 to 68 Ma)^10,11^.

Existing geophysical and geological data provide some constraints on the ophiolite deep structure. Previous gravity anomaly modelling^7,8^ suggest the ophiolite is ~5–8 km thick and the present day Moho depth is 30-40 km. Seismic data^12^ suggest a deeper Moho (~45 km) and possible thrust repetitions of the shelf carbonates beneath the ophiolite. Finally, field mapping reveals a distinct thrust slice of high-temperature granulite facies rocks brought up by an out-of-sequence thrust within the mantle sequence of the ophiolite, raising the possibility that the crust beneath the ophiolite may presently comprise stacked units of Late Cretaceous granulites, similar to the Bani Hamid thrust sheet^11^.

Despite these previous studies, the sub-surface geometry of the Oman–UAE ophiolite remains poorly known because previous gravity models are unconstrained seismically and seismic reflection profile data have difficulty in imaging its internal structure. Major tectonic questions include the nature of the crust that underlies the ophiolite, its easterly offshore extent and the relationship of the ophiolite to the crust and mantle of the Gulf of Oman.

Here, we report the results of an onshore/offshore seismic experiment in the UAE that addresses these questions by a combination of active and passive seismic techniques, potential field modelling and surface geological mapping. We find the east-dipping Semail ophiolite is >15 km thick, flexed down the pre-existing crust by >5 km, is presently underlain by folded and thickened continental crust and is bound to the east by a major normal fault, implying the ophiolite may not be rooted in the Gulf of Oman crust and mantle.

## Results

### Geophysical data

**Fig. 1:**
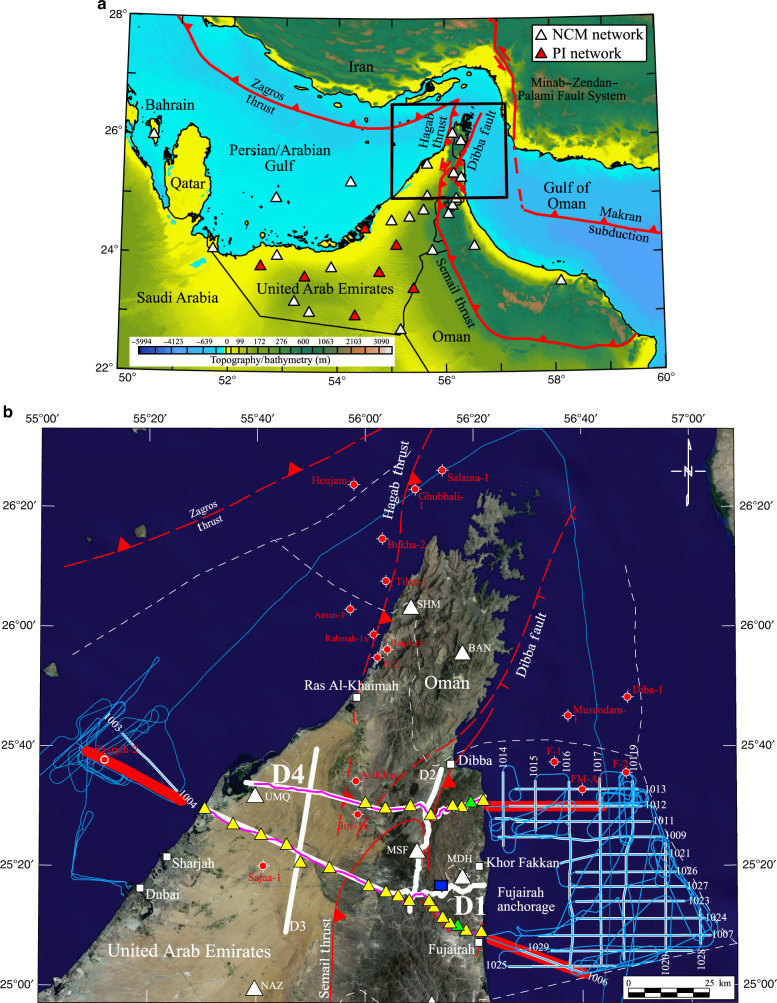
Location map showing the transects along which geological and geophysical data were acquired. **a** Study area (black box) and permanent passive seismic stations (white and red filled triangles). Topography/bathymetry data based on Becker et al.^42^. **b** Landsat 9 image (https://www.geosoft.com/products/dap-server/overview) with geophysical data. Offshore: thick white lines = seismic reflection profiles; thin blue lines = gravity and magnetic anomaly data. Onshore: thick white lines = vibroseis seismic reflection profiles^12^. Thin purple lines = gravity and magnetic anomaly data. Yellow filled triangles = Petroleum Institute (PI) seismic broadband stations. White filled triangles = National Centre of Meteorology (NCM) stations. Green filled triangles = Stations S-15 (D1) and S-19 (D4). Blue filled square locate the Bani Hamid granulites^11^. Thin solid red lines delineate the Late Cretaceous Semail thrust and the Oligocene-early Miocene Hagab thrust and the Dibba fault^20,43^. Dashed where uncertain. Figures constructed using Oasis Montaj (https://www.geosoft.com/products/oasis-montaj).

**Fig. 2:**
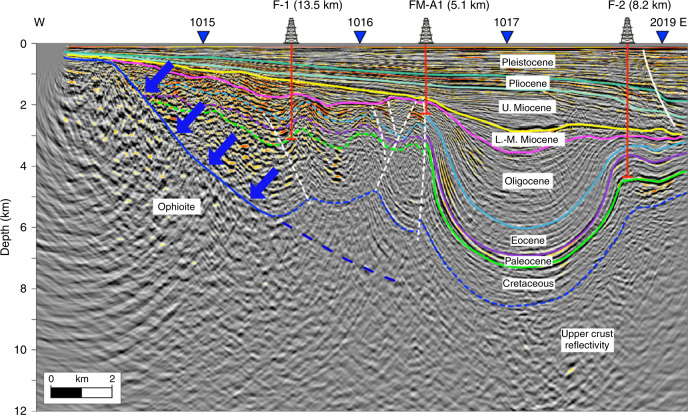
Depth converted seismic reflection Line 1012, which extends Transect D4 offshore. The Line is located in Fig. 1. Blue filled arrows delineate the top of the ophiolite, the surface of which has acted as a major normal fault. Prominent reflectors (solid coloured lines) define the main stratigraphic units, the age of which has been determined on the basis of F-1, FM-A1 and F-2 well ties. Yellow line marks a major unconformity. Dashed and solid white lines show faults. Dashed coloured lines show uncertain correlations. The uninterpreted reflection Line 1012 together with Line 1006, which extends Transect D1 offshore, are shown in Supplementary Figs. 1 and 2.

During June/July 2014 we carried out an active source seismic experiment that included 22 multichannel seismic reflection profiles offshore (Lines 1003–1019, Fig. 1) and 2 wide-angle (refraction) transects onshore/offshore (Transects D1, D4, Fig. 1). The onshore segment followed Vibroseis lines previously acquired by the Ministry of Energy, UAE in 2003^12^.

Figure 2 shows an example of a depth migrated stack on Line 1012, which extends Transect D4 to the east (Fig. 1). The stack reveals a major unconformity that separates gently dipping sediments above from structurally deformed sediments below which we interpret as of Middle/Late Miocene age on the basis of well ties. Below the unconformity, sediments have been deformed into a narrow synclinal structure and at the western end of the profile they appear to terminate on seismic basement. We interpret seismic basement as top of the ophiolite. The surface separating the ophiolite from the overlying sediments dips gently seaward, increasing to ~40–45^o^ at depth (Fig. 2), and we interpret it as a major normal fault.

**Fig. 3:**
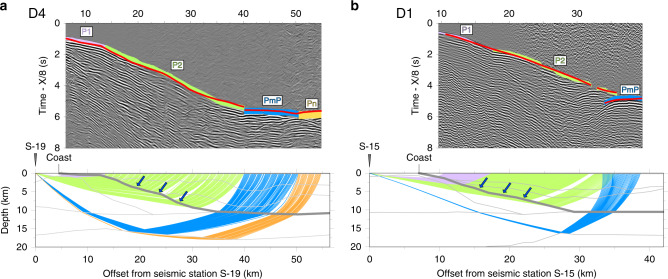
Examples of receiver gathers from onshore seismic recording stations and shots at sea. The record sections have been reduced to a velocity of 8 km s^−1^ with observed (coloured lines) and predicted (thin red line) intra-crustal refractions (P1, P2), possible Moho reflections (PmP) and possible mantle refractions (Pn). Thickness of coloured line is proportional to travel time error, based on source-recording station offset. Ray coverage plot showing paths for the selected picked arrivals. Inverted triangle = station location. Blue arrows show the surface with abrupt velocity contrast, interpreted as top of the ophiolite. Grey lines show layers used during modelling of observed traveltimes. **a** Station S-19 on Transect D4 (Fig. 1). **b** Station S-15 on Transect D1 (Fig. 1). The uninterpreted record sections are shown in Supplementary Fig. 3.

Figure 3 shows an example of an onshore receiver gather, a ray coverage plot of the main arrivals and a velocity model derived from rayinvr^13^. Two distinct P wave velocity domains were imaged either side of the coast. To the east, the upper crust is dominated by relatively low velocities in the range ~2–5 km s^−^^1^. We interpret these velocities as sediments which progressively thicken towards the offshore, reaching a maximum thickness of around 10 km. Seismic velocities then increase sharply from 5.0 to 6.5 km s^−1^ below the sediment layer (45–50 km model distance) to 7.1–7.2 km s^−1^ until they appear to reach Moho at about 18 km depth. To the west, the upper crust is dominated by velocities in the range ~5–6 km s^−1^, typical of sheeted dikes or upper gabbros from ophiolites in Troodos (Cyprus), Semail (Oman), Papua-New Guinea and Bay of Islands (Newfoundland)^14^. Velocities decrease abruptly to the east, where they trace out an east-dipping surface that separates the ophiolite from sediments (Fig. 3, blue arrows) and increase to the west, towards the mountains, indicating more mafic material although we caution the ray coverage is limited here and so it is not possible from refraction data alone to define the western limit of the ophiolite.

The crust beneath the ophiolite exhibits gradually increasing velocities but reveals extremely high velocities at the base where velocities up to 7.6 km s^−^^1^ are recorded. The Moho appears to gently bulge at middle model distances but upper mantle type velocities are comparable with those found at larger model distances.

**Fig. 4:**
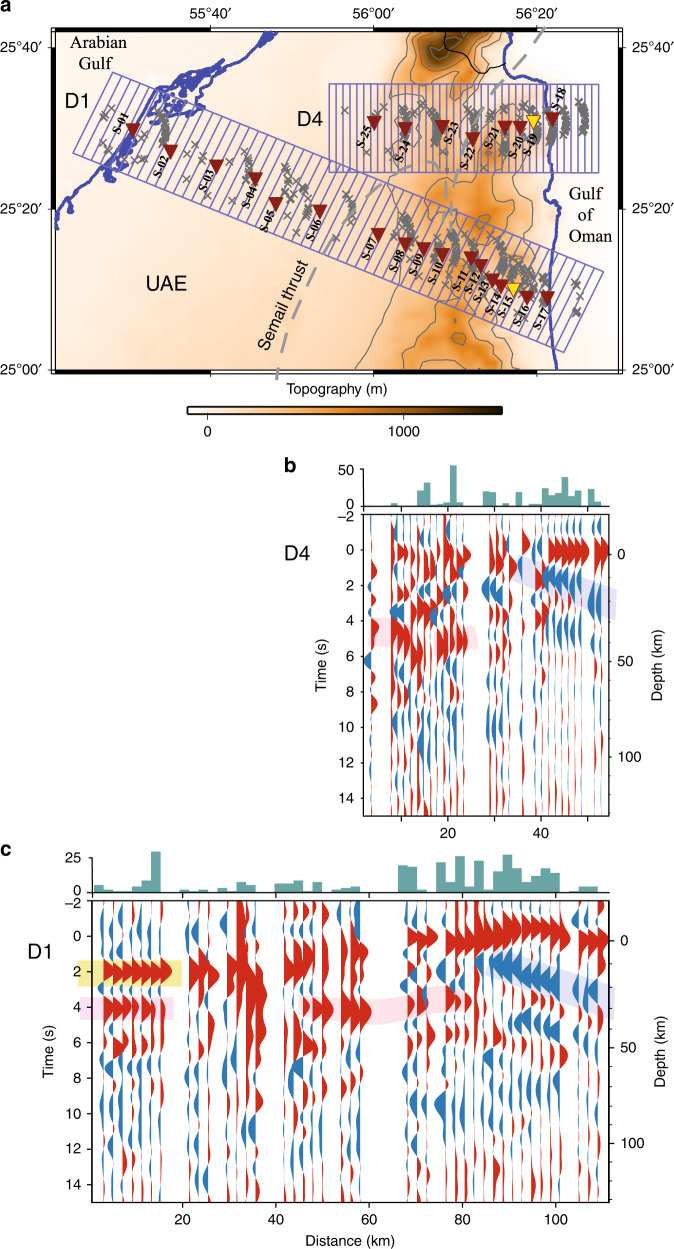
Receiver function profiles along Transects D4 and D1. **a** Location map showing brown shaded topography. Maroon filled inverted triangles = station locations. Yellow filled inverted triangles = stations S-15 and S-19 (Fig. 3). Boxes delineate the 2 × 20 km (D4) and 1.4 × 20 km (D1) bins used to group the data and projected piercing point locations at 30 km depth are marked by a grey ×. **b** Receiver function profiles along Transect D4 showing the stacked trace from each bin with red indicating positive polarity and blue negative. Trends discussed in the text are highlighted by coloured background shading. Histograms above the profiles show the relative number of traces contributing to each stack. Depth conversions displayed on the right have been computed using the iasp91 velocity model^44^. **c** Receiver function profiles along Transect D1.

Broadband seismometers deployed along Transects D1 and D4, together with a seismic network operated by the NCM, were also used to record over 2 years of passive seismic data. This data set was used to calculate receiver functions from teleseismic earthquakes of moment magnitude (Mw) >5.5 within epicentral distance 30–98^o^. Depth points were then obtained from these functions using the *H*–*k* stacking method^15^ which performs a grid search across a range of depths (*H*) and Vp/Vs values (*k*) to find the best fits to the projected arrivals of the Ps, PpPs and PpSs/PsPs phases. A Moho signal was obtained west of the mountains at ~4 s (pink shading, Fig. 4b, c), and mapped to a depth of ~36 km with *H*–*k* stacking. A strong positive polarity signal at 2 s (yellow shading, Fig. 4b, c) was also obtained and interpreted as a conversion from the relatively high velocity underlying the Arabian basement to the relatively low velocity overlying foreland basin sediments. Finally, a strong, eastward dipping negative polarity signal from 2–4 s (blue shading, Fig. 4b, c) across west of the mountains was obtained and interpreted as a conversion from the relatively low velocity underlying obducted Tethyan passive margin sediments to the relatively high velocity overlying obducted Semail ophiolite. This interface has an easterly dip, similar to that of the seismically imaged top of the ophiolite, and we believe it defines the base of the ophiolite.

### Gravity and magnetic anomaly modelling

Gravity and magnetic anomaly data were used to construct a density and susceptibility model along Transects D4 and D1 (Fig. 1). Bouguer anomalies increase from near zero values at the western limit of the onshore mantle ophiolite to maximum values of ~170 mGal over the crust ophiolite at the coast. Magnetic anomalies are subdued over the onshore mantle ophiolite outcrop, but increase towards the coast, and offshore there is a ~12 km wide belt of high amplitude short wavelength magnetic anomalies^16^ that we believe reflects the seaward extension of the crust ophiolite.

Modelling was carried out using a two-dimensional line-integral method of calculating the gravity and magnetic effect of undulating interfaces of constant density contrast^17^. The structure along the transect was sub-divided into a number of bodies of uniform density and the density contrasts calculated between the bodies and a zero elevation reference column where it is assumed gravity anomalies average to zero. We used the same bodies as deduced in the gravity modelling in the magnetic anomaly modelling, assigning the ophiolite bodies a uniform susceptibility of 0.00065 to 0.00130 SI units and all other bodies a zero-uniform susceptibility. We only considered induced magnetisation so, ignored the possibility of remanent magnetisation and its contribution to the total magnetisation vector.

**Fig. 5:**
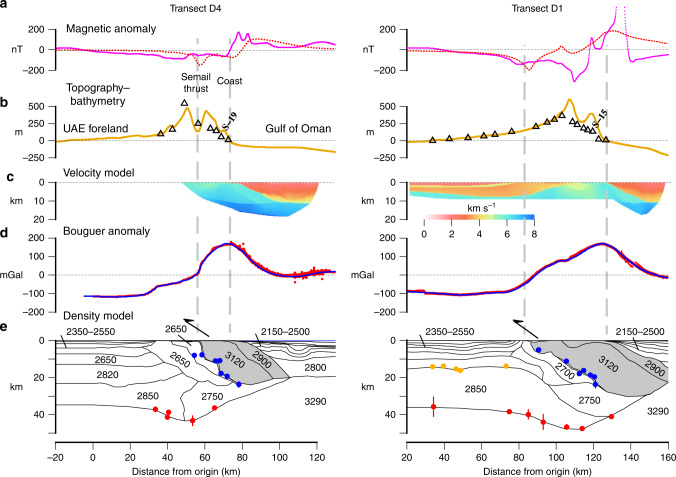
Comparison of observed and calculated gravity and magnetic anomalies along Transects D4 and D1 (Fig. 1). The data along the transects have been projected on to two different profiles: for transect D4 we used an azimuth of 90.0^o^ and an origin located at 55.64^o^E and 25.50^o^N and for profile D1 we used an azimuth of 113.5^o^ and an origin located at 55.20^o^E and 25.61^o^N. **a** Comparison of observed magnetic anomaly (solid purple lines) to the calculated anomaly (red dotted line) assuming the same ophiolite bodies that cause the gravity anomaly also cause the magnetic anomaly (grey shaded in **d**). **b** Topography/bathymetry profile (solid brown line) showing the location of the onshore seismic stations. **c** Seismic P wave velocities derived from sea shots recorded at the stations. **d** Comparison of observed Bouguer anomaly (red points) and calculated anomaly (solid blue line) based on the bodies outlined in **e**. **e** Crustal model showing densities assumed in the calculation of the gravity anomalies. Filled red (Moho) and blue (base ophiolite) circles: selected depth converted receiver function positive (velocity increase with depth) and negative (velocity decrease with depth) polarity events respectively. Figure constructed using GMT^45^.

Figure 5 shows the body configurations, together with their respective densities, that generally explain the observed gravity and magnetic anomalies and are in general accord with the top and base of the ophiolite as defined by seismic data. The fit between observed and calculated Bouguer anomalies is particularly good. Models reveal a region of high density at the eastern end of Transects D4 and D1 and the western end of Lines 1012 and 1006 that is flanked on either side by a region of low density. The highest density body of 3120 kg m^−^^3^ intersects the surface at 55–72 km along D4 and at 95–127 km along D1 where it corresponds to a mapped dunite outcrop. The lowest density bodies of 1950–2300 kg m^−3^ occur in the flanks of the Oman–UAE mountains and correspond to sediments which have infilled the UAE foreland basin to the west and the hinterland basin underlying the Gulf of Oman to the east. The fit between the observed and calculated magnetic anomalies is poor, but a magnetic anomaly high over the centre of the mantle ophiolite which is flanked to the east by a broad low is a characteristic feature of both observed and calculated magnetic anomalies.

The top of the ophiolite imaged as a fault bounded surface on reflection Lines 1012 and 1006 (Fig. 2, Supplementary Fig. 2) and as a prominent refractor for sea shots at stations S-19 and S-15 (Fig. 3) correlates with a steep decrease in the observed Bouguer anomaly, and our modelling suggests a density contrast across it of at least 500–850 kg m^−3^.

The density and susceptibility values used in the modelling are in general accord with previous studies. For example, the 2800 and 3120 kg m^−3^ we assume for the ophiolite oceanic crust and ophiolite mantle respectively are compatible with the densities based on laboratory measurements of oceanic crust along the Ibra-Muscat transect of the Oman ophiolite^18^. Moreover, the value of 3120 kg m^−^^3^ we assume for ophiolite mantle is intermediate between those deduced^18^ for serpentinised (2790 kg m^−3^) and unserpentinised (3300 kg m^−3^) mantle, indicating some degree of serpentinisation (~35%) for the Semail ophiolite, at least along the transects. The low P wave velocities derived west of the coast in the refraction part of the experiment are consistent with serpentinisation, although they are lower (by up to ~1 km s^−^^1^) than would be expected for ~35% serpentinisation^19^.

## Discussion

The Semail ophiolite sequence is a regular Penrose-type ophiolite with a harzburgite–dunite mantle sequence, with a 1–2 km thick Moho transition zone comprising interbanded harzburgites, dunites and wehrlites with overlying gabbro sill complexes intruded by tonalite–trondhjemite dykes, and an upper crust comprising sheeted dykes and extrusive basalts^20–22^. From structurally lowest to highest position, the allochthonous sheets, include the Sumeini Group, comprising shelf-edge and slope-carbonate sediments; the Hawasina Complex, with distal-slope and deep-sea Tethyan sediments; the Haybi Complex, comprising Mesozoic exotic limestones (Oman Exotics), volcanics (Haybi volcanics), mélanges and sub-ophiolitic metamorphic rocks; and the Semail ophiolite complex. Regionally, in the northern Oman and UAE mountains, the Semail ophiolite and subjacent allochthons dip to the E-NE^23^. Seismic and gravity modelling (Fig. 5d) confirm the broad sub-division of the geological units onshore, and reveal the steepness at depth of the eastward regional dip (~40–45^o^) of both the allochthonous sheets and the main crust and mantle ophiolite bodies.

**Fig. 6:**
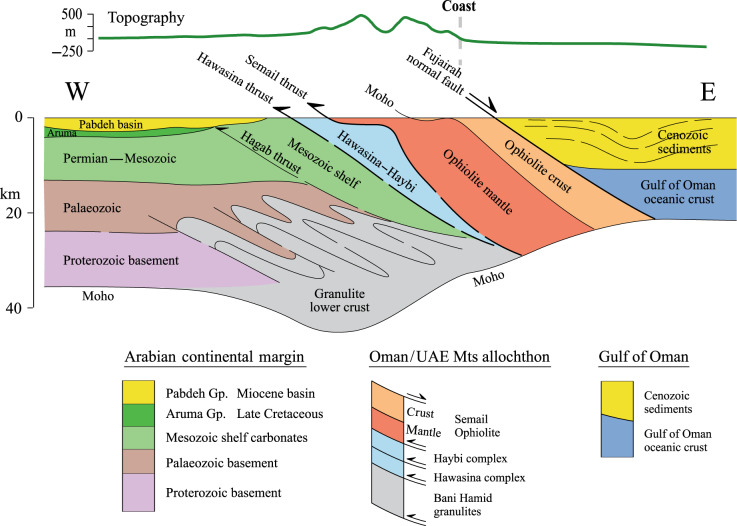
Summary topography profile and geological cross-section with interpretation. The cross-section is consistent with both geological field observations and the results of geophysical (seismic, gravity and magnetic) modelling of data acquired along the Transects D4 and D1 of the UAE shown in Fig. 1.

Figure 6 shows the allochthonous sheets, the main ophiolite bodies, and the configuration of the underlying continental crust and flanking oceanic crust onshore and offshore the UAE that are consistent with geological field observations and geophysical modelling. The nature of the crust is unclear but it has clearly been thickened and depressed beneath the allochthonous sheets and ophiolite. The upper crust beneath the mountains comprises Tethyan margin shelf sediments, but basement is not exposed in the UAE. Well data (e.g. from the Shah field), together with gravity and aeromagnetic modelling^16^, suggest a Proterozoic basement that was deformed during a Carboniferous–early Permian orogenic event. The lower crust is interpreted here as comprising Late Cretaceous high-temperature granulite facies meta-carbonate and meta-quartzite rocks, similar to rocks exposed in the Bani Hamid thrust sheet in the UAE^11^. The presence of these rocks is consistent with the densities modelled in Fig. 5e (2750 kg m^−3^) and those measured from 30 global samples of calcite marbles (2743 ± 14 kg m^−3^)^24^.

The thickness of the ophiolite sequence measured stratigraphically from Fig. 5d is up to ~10–20 km, which is in general accord with previous estimates, for example those based on field mapping^20,25,26^ and gravity data^18^. Also, in accord are observations in Papua-New Guinea^27,28^ where the Bouguer gravity anomaly is in the range 100–150 mGal and the ophiolite is 12–16 km thick and in the Troodos massif^29^ where the Bouguer gravity anomaly is in the range 100–150 mGal and the ophiolite is ~14 km thick. The Bouguer anomaly along Transects D4 and D1 is greater in amplitude (~170 mGal) than both Papua-New Guinea and Troodos, consistent with our somewhat thicker estimate for the ophiolite.

Gravity modelling (Fig. 5e) and geological interpretation (Fig. 6) suggest that following rifting and thinning during the Triassic and Early Jurassic, the Arabian plate crust beneath the Oman–UAE mountains was significantly thickened by thrusting and folding. At 95 Ma, post-rift subsidence was interupted by ophiolite obduction, when the crust was folded and thickened as the ophiolite and underlying thrust sheets were emplaced onto the continental crust. Isoclinal folds in the Bani Hamid granulites are testament to the crustal thickening that occurred in the lower crust granulites during the Late Cretaceous.

Isostatic considerations suggest that ophiolite obduction would have resulted in a significant loading and subsidence of the pre-existing Arabian crust^4,7,8^. Although modified during the subsequent folding and thrusting, evidence for this subsidence may still exist in the crustal structure and the gravity anomaly. To test this possibility, two-dimensional process-oriented flexure and gravity models^30^ have been constructed for ophiolite loading of a pre-existing rifted continental margin (Supplementary Fig. 4). We assume in the models that the driving ophiolite loads displaced water, the pre-existing margin had a maximum slope and depth of 45^o^ and 5 km, respectively, the density of the material infilling the flexure varied spatially, and the elastic thickness of rifted crust and lithosphere was small, and ~5 km. The shape and densities of the driving loads were estimated from Fig. 5c, d. Supplementary Figure 4 shows that ophiolite loading could contribute >5 km of subsidence of a pre-existing rifted margin. In addition, the models predict the maximum downward deflection of Moho is offset landward of the ophiolite body and a broad positive gravity anomaly, similar to the observations in Fig. 5d, e.

A common feature of the seismic data and gravity and magnetic models off the east coast of the UAE between Fujairah and Dadna is a major east-dipping normal fault. The fault surface is recognised as a steeply dipping reflector (Fig. 2) and refractor (Fig. 3) and we believe it represents a major through-going fault that separates the Arabian crust and its allochthonous sheets to the west from oceanic crust and mantle beneath the Gulf of Oman to the east. While our gravity and magnetic models do not show a contrast in either density or susceptibility beneath the seismically defined fault, such a contrast would not be expected given that it may bring into contact Gulf of Oman oceanic crust with the Tethyan oceanic crust that comprises the Semail ophiolite. We interpret this fault to mark the eastern boundary of underthrust Arabian continental crust beneath the ophiolite.

This interpretation implies that the Semail ophiolite may not be rooted in the present day Gulf of Oman and that the normal fault represents the boundary of a major incipient suture that is forming during the earliest stage of Arabia–Makran plate collision. The fault would have been reactivated as a normal fault following ophiolite emplacement, thereby forming the broad depositional hinterland sedimentary basin that now underlies the Gulf of Oman. Seismic and well data suggest the basin rapidly deepened during the Late Cretaceous as a consequence of ophiolite loading, concomitant with deepening of the flexural Aruma foreland basin west of the Oman–UAE mountains^6,31^. The basin continued to deepen throughout the Palaeocene–Early Miocene, a time of thick-skinned thrusting in the basement and cover units^32^ and the culmination of the Musandam mountains^31^, during which time it was infilled by deep-water shales and mudstones.

An outstanding question is the nature of the crust that underlies the present day Gulf of Oman. We have interpreted this crust to be oceanic and three-dimensional flexural backstripping^33^ of sediment isopachs reveals a tectonic subsidence similar to what would be expected for Late Cretaceous oceanic crust. However, backstripping reveals the tectonic subsidence associated with all the loads that act on the crust, including those due to ophiolite loading and so the oceanic crust could be significantly younger than Late Cretaceous. Presently, the Gulf of Oman oceanic crust is subducting beneath the Makran accretionary wedge to the north^34^, offshore and onshore Baluchistan. The suture zone may therefore represent an incipient plate boundary that could evolve into a structure similar to the Zagros suture in Iran^35^ and eventually into a structure similar to the Indus–Tsangpo suture zone that marks the collision between the Indo-Australian and Eurasian plates^36^.

## Methods

### Data acquisition

The geophysical data used in this paper were acquired onshore in the UAE and offshore in the Arabian Gulf and Gulf of Oman on SeaBird Exploration’s *M/V Hawk Explorer*. The Hawk Explorer was equipped with a 4 × 12 element air gun array, a 5 km long streamer, a Lacoste-Romberg air-sea gravimeter, and a sea-spy proton precession magnetometer. During the refraction experiment a 7060 cu. in. (116 l) air gun array was fired at 50 s intervals. Refracted and reflected phases were recorded onshore on 18 Nanometrics Posthole 120 and 7 Guralp 3ESPCD (on loan from SEIS-UK) three-component broadband seismic stations, deployed at ~15 km interval along Transects D1 and D4. During the reflection experiment a 5460 cu in (90 l) air gun array was fired at 25 s interval and reflected arrivals were recorded on the 48-channel digital streamer. Gravity and magnetic data were acquired continuously along all seismic profiles. Onshore gravity data were acquired at 149 stations along Transect D1 and D4 with a Scintrex CG-5 Autograv gravimeter.

### Seismic data processing and modelling

Seismic data were processed using standard techniques. Reflection data were processed using ProMax and Seismic Unix and techniques such as semblance, spherical convergence and deconvolution. Particular problems encountered during processing were inter-bed, short and long period multiples, reverberations in shallow water, turning noise, seismic interference and cable noise. Incorporation of receiver and source side de-ghosting helped to improve resolution both at the low- and high-frequency end of the spectrum. WesternGeco kindly provided final pre-stack processing and conversion of Two-Way Travel Time data to depth.

Refraction profile data were processed using GLOBE Claritas and techniques such as shot extraction, sorting to receiver gathers, filtering, and Automatic Gain Control. Travel times of different phases were picked manually. As the land seismic stations were continuously recording during the experiment, we used the exact timing of the air gun firing to cut the seismic records into separated traces of 40 s length and collected them into receiver gathers. After conversion to SEGY, the processing included the application of frequency filtering using a band-pass Butterworth filter (2–4–13–15 Hz), automatic gain control and a coherency filter to enhance the visibility of different phases. We used a simple starting velocity model in rayinvr (http://terra.rice.edu/department/faculty/zelt/rayinvr.html) incorporating information from seismic reflection profile data and carried out both iterative forward modelling and inversion in order to best account for the observed picks, adjusting the starting velocity model as required. Topography was taken into account (included in the SEGY headers during transformation of mini-seed data to SEGY) and also when modelling the travel times in rayinvr. Data quality is variable but generally good, allowing clear arrival identification up to about 70 km offset.

### Receiver function analysis

Receiver function analysis was carried out using data from the United States Geological Survey global earthquake catalogue, a network of closely spaced onshore recording stations and the core codes in https://github.com/bkeats/Crustal-structure-ophiolites-and-flexure-beneath-the-Oman-UAE-mountains. After testing a range of search parameters, all earthquakes within a range of 30–98°, and with a moment magnitude Mw > 5.5 were selected, as smaller events consistently failed signal-to-noise ratio tests. A total of 382 earthquake events were recorded during July 2014 and November 2016, with a bias to the east due to the number of large magnitude events occurring in the subduction zones of the northeast Indian and western Pacific oceans. Not every event was recorded at each station. The majority of data processing and analysis was carried out using ObsPy^37^ and rf^38^. Prior to analysis we (a) calculated projected arrival times for the primary P phase (tP) of each event using the TauP module in ObsPy, (b) cut the raw seismograms in a 200 s initial analysis window centred around the projected arrival time tP and (c) de-trended, de-meaned, downsampled data to 25 Hz, and applied a Butterworth band-pass filter to restrict the frequency content to between 0.05 and 4 Hz to limit artifacts. Receiver functions were then calculated using either the multi-taper correlation^39^ or time-domain deconvolution^40^ methods. Results at each station were inspected for variations with back azimuth and epicentral distance and grouped accordingly for *H*–*k* stacking^15^. An average crustal velocity of 6.5 km s^−1^ was used for depth conversion; however, this value was decreased slightly for shallow phases. Weights were generally set at 0.5, 0.3 and 0.2 for the Ps, PpPs and PsPs/PpSs phases, respectively. The *H* search ranges were initially set to broad crustal windows, and later reduced to narrower windows around the potential interface depths with an increment of 0.2 km. The *k* search range was typically set at 1.6–1.9 with an increment of 0.01. For bootstrapping error estimation the search space was further restricted so to improve computation time and avoid the *H*–*k* algorithm jumping to alternative sets of phases. With this method we applied the *H–k* stacking algorithm to each subset of receiver function results, and repeated the process across a range of *H* and *k* windows to extract depth points at each station. For some results a strong primary phase could be matched to several potential multiples: typically one with a high *k* and one with a low *k*. In this case, the preferred result was typically selected by favouring high *k* at shallow depth (and vice versa), by comparing the results to those at nearby stations, or to those calculated using different deconvolution methods.

### Gravity and magnetic data processing and modelling

Gravity and magnetic anomaly data were processed using standard techniques. Offshore data were corrected for Eötvos, cross-coupling and instrument drift, tied into gravity base stations in Port Dubai and Fujairah, and processed through to free-air gravity anomalies. The data were corrected for mass and height above the geoid and for terrain and were processed through to Bouguer anomalies. Gravity modelling was based on a reference column comprising a zero elevation 31.2 km thick, 2754 kg m^−3^ density continental crust overlying a 93.8 km thick 3236 kg m^−3^ density mantle which is in isostatic balance with a mid-ocean ridge column comprising a water depth of 2.5 km, a 5 km thick 2737 kg m^−^^3^ density oceanic crust and a 117.5 km thick 3176 kg m^−3^ density mantle. Offshore the magnetic data were corrected for diurnal and secular variation. Onshore we used an aeromagnetic database^16^.

## Supplementary information

Supplementary Information

## Data Availability

The underway geophysical data (gravity, magnetics and bathymetry) acquired on *M/V Hawk Explorer* (Fig. 1) and used in Fig. 5d will be deposited in the Marine Geoscience Data System (see http://www.geomapapp.org/index.htm). SEGY files of the depth migrated stacks along Line 1012 and Line 1006 shown in Fig. 2 and Supplementary Figs. 1 and 2 are available from the authors. Passive seismic data acquired by the NCM can be obtained by contacting the NCM^41^. Data acquired by the PI and used in Figs. 3 and 4 will be made accessible through the IRIS Data Management (http://www.iris.edu/mda) from June 2021 (see 10.7914/SN/4K_2014)^41^.
