# Supplementary material for: Geophysical imaging of ophiolite structure in the United Arab Emirates
Source: Nat Commun. 2020 May 29;11:2671. doi: 10.1038/s41467-020-16521-0 (PMC7260221; doi:10.1038/s41467-020-16521-0)
Supplement: Supplementary file 1 — Supplementary Information [file 41467_2020_16521_MOESM1_ESM.pdf]

Supplementary Information

Geophysical imaging of ophiolite structure in the United Arab Emirates

M. Y. Ali[1], *A. B. Watts[2], M. P. Searle[2], B. Keats[2], S. Pilia[1,3], T. Ambrose[2]

[1]Khalifa University of Science and Technology, Abu Dhabi, UAE

[2]Department Earth Sciences, University of Oxford, South Parks Road, Oxford OX1 3AN, UK

[3]Department of Earth Sciences, University of Cambridge, Cambridge, CB3 OEZ, UK

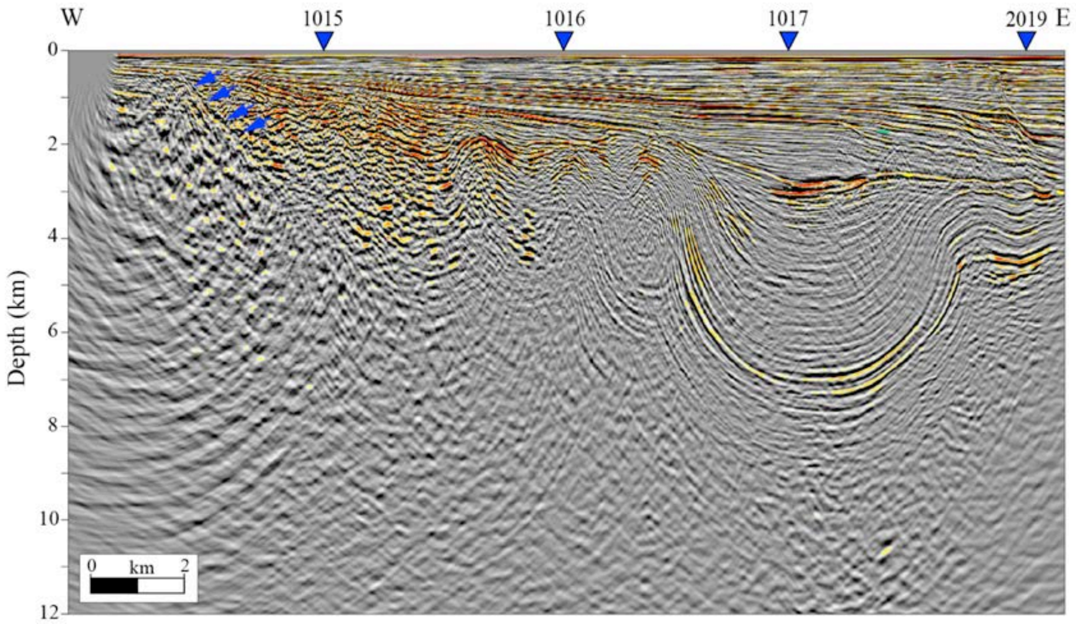

Supplementary Figure 1. Uninterpreted depth converted seismic reflection Line 1012 on Transect D4 (Fig. 2). Blue arrows identify the top of the ophiolite. Inverted blue filled triangles show points of intersection with other multichannel seismic reflection lines acquired during the survey of M/V Hawk Explorer.

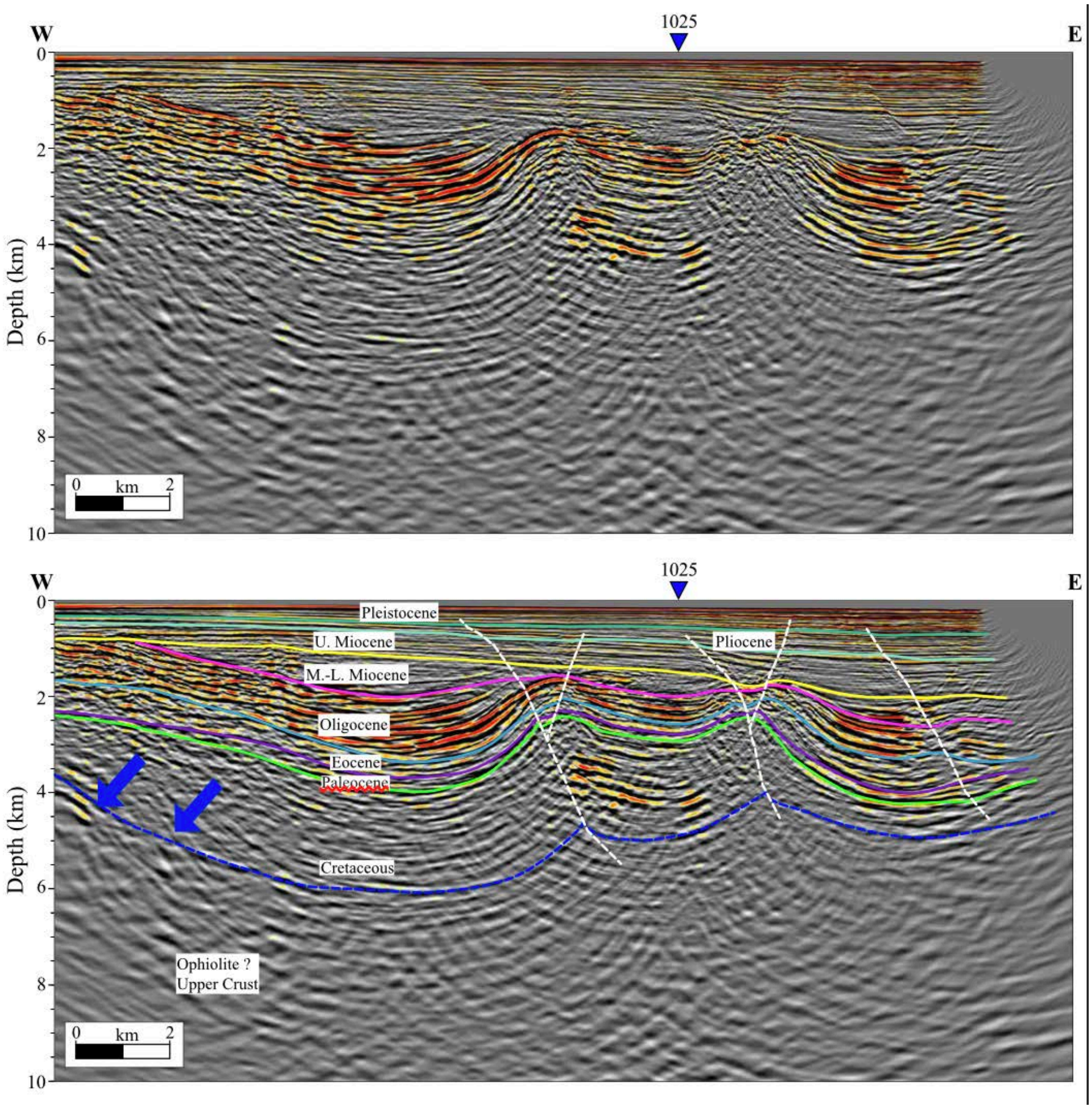

Supplementary Figure 2. Uninterpreted and interpreted depth converted seismic reflection Line 1006 on Transect D1 (Fig. 1). Blue arrows identify the top of the ophiolite. Inverted blue filled triangles show points of intersection with other multichannel seismic reflection lines acquired during the survey of M/V Hawk Explorer.

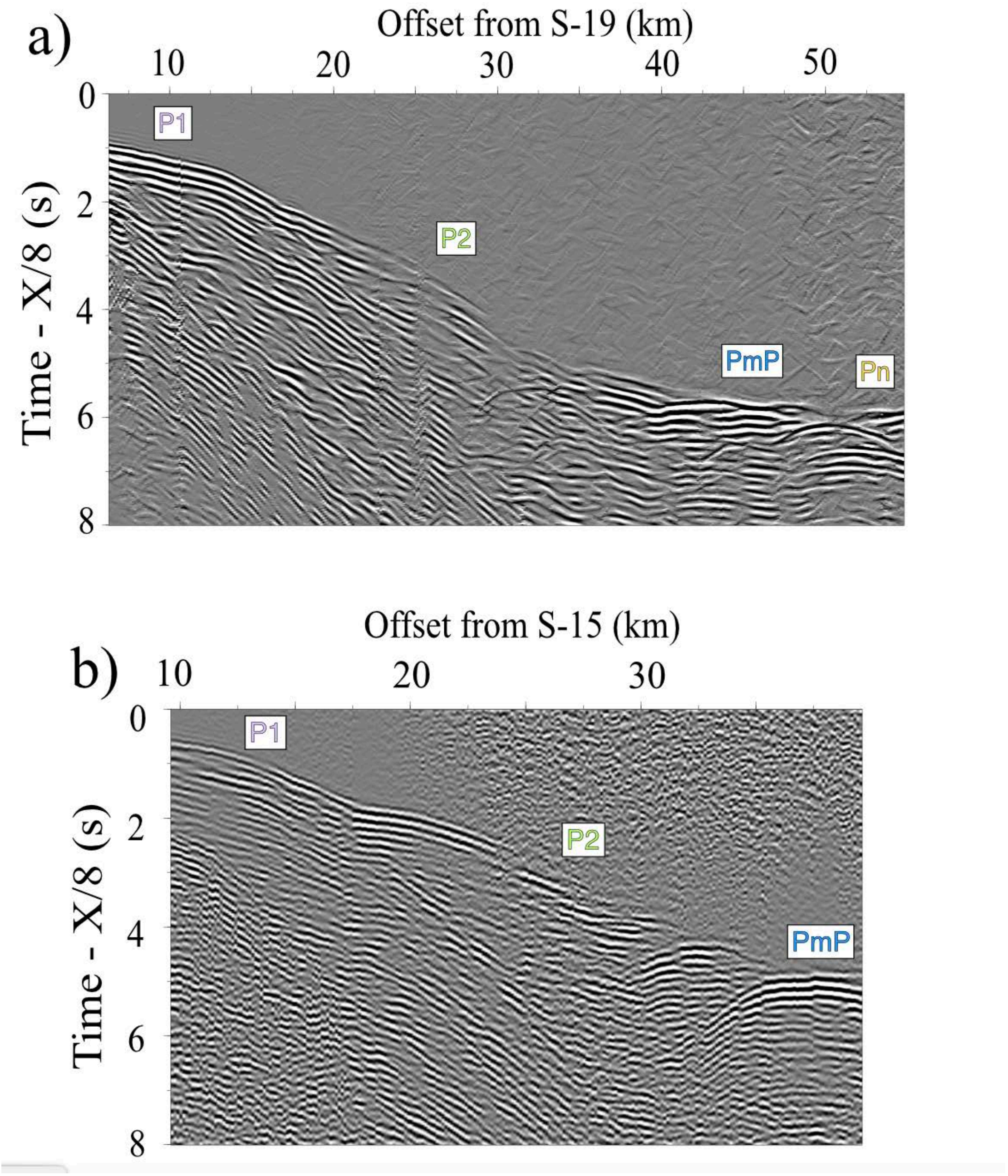

Supplementary Figure 3. Uninterpreted wide-angle receiver gathers from the vertical component of seismic station S-19 on Transect D4 and S-15 on Transect D1 (Fig. 1). The record section has been reduced to a velocity of 8 km s^{-1}.

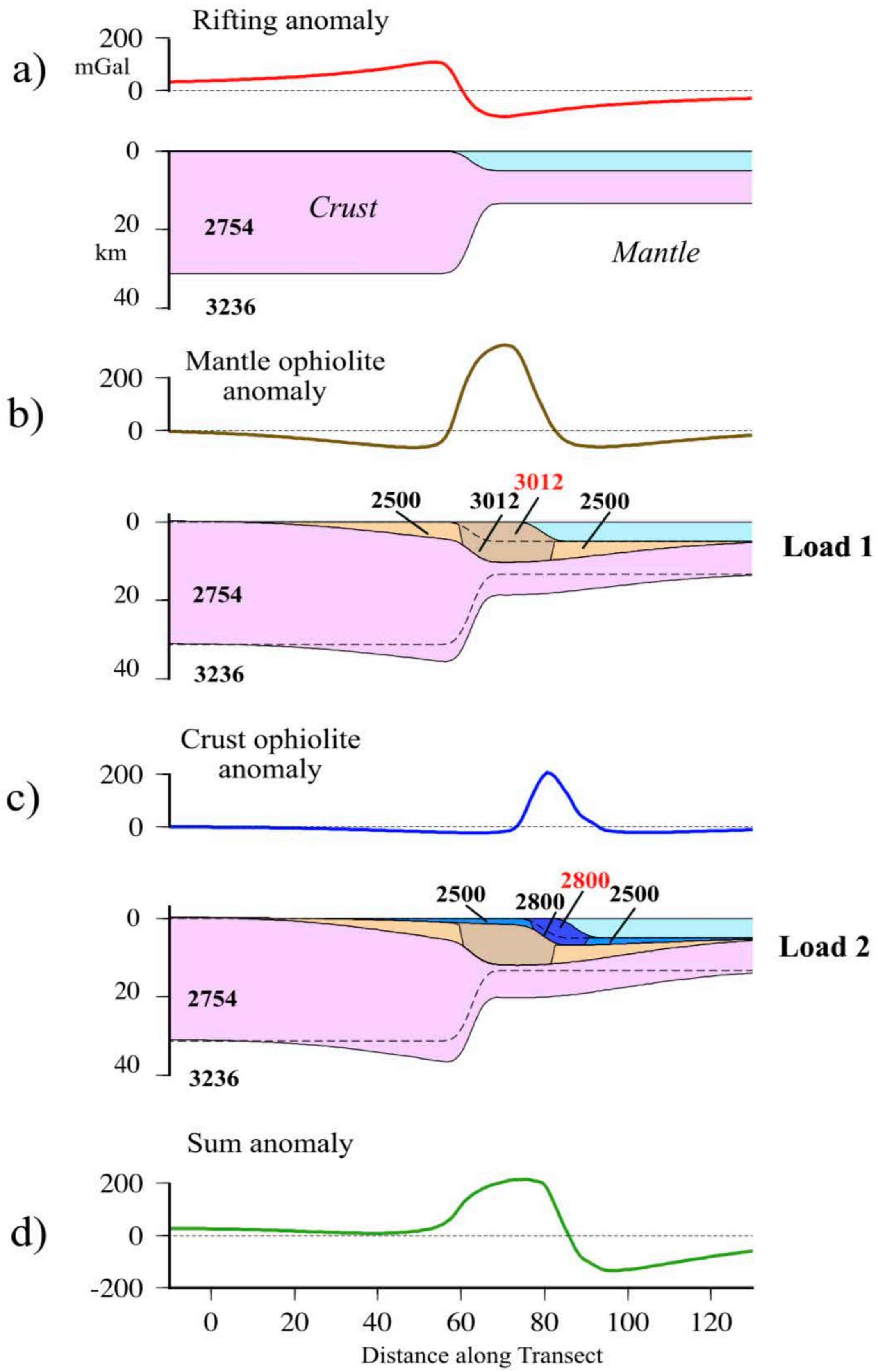

Supplementary Figure 4. Simple model of flexure for crust and mantle ophiolite loading of a pre-existing rifted continental margin with a spatially varying elastic thickness, T_e. a) Rifting gravity anomaly and crustal structure. b) Mantle ophiolite

gravity anomaly and crustal structure. c) Crust ophiolite gravity anomaly and crustal structure. d) Sum gravity anomaly obtained by adding the rifting, mantle ophiolite and crust ophiolite anomalies. Numbers in bold indicate densities assumed for the crust and mantle and for the driving (red font) and infill (black font) loads in kg m^{-3}. The driving load is caused by the displacement of water by the ophiolite and the infill load is caused by the material (assumed here to be ophiolite and its mass wasting products that infills the flexure. The flexure has been computed assuming a T_e = 5 km beneath the ophiolite and a T_e = 20 km in flanking regions. No attempt has been made here to include the effects of post-ophiolite obduction sedimentation in the UAE foreland and Gulf of Oman, which will modify both the sum gravity anomaly and the crustal structure. The figure illustrates that combined crust and mantle ophiolite loading can flex downwards a pre-existing rifted margin by 10 km or more.
